# Soft-tissue perineurioma of the retroperitoneum in a 63-year-old man, computed tomography and magnetic resonance imaging findings: a case report

**DOI:** 10.1186/1752-1947-4-290

**Published:** 2010-08-26

**Authors:** Mayumi Yasumoto, Yoshiaki Katada, Reiko Matsumoto, Akiko Adachi, Koh Kaneko

**Affiliations:** 1 Departments of Radiology, Saitama Red Cross Hospital, 8-3-33, Kamiochiai, Chuo-ku, Saitama, Saitama, Japan; 2 Department of Pathology Saitama Red Cross Hospital, 8-3-33, Kamiochiai, Chuo-ku, Saitama, Saitama, Japan

## Abstract

**Introduction:**

Soft-tissue perineuriomas are rare benign peripheral nerve sheath tumors in the subcutis of the extremities and the trunks of young patients. To our knowledge, this the first presentation of the computed tomography and magnetic resonance imaging of a soft-tissue perineurioma in the retroperitoneum with pathologic correlation.

**Case presentation:**

A 63-year-old Japanese man was referred for assessment of high blood pressure. Abdominal computed tomography and magnetic resonance imaging showed a well-defined, gradually enhancing tumor without focal degeneration or hemorrhage adjacent to the pancreatic body. Tumor excision with distal pancreatectomy and splenectomy was performed, as a malignant tumor of pancreatic origin could not be ruled out. No recurrence has been noted in the 16 months since the operation. Pathologic examination of the tumor revealed a soft-tissue perineurioma of the retroperitoneum.

**Conclusion:**

Although the definitive diagnosis of soft-tissue perineurioma requires biopsy and immunohistochemical reactivity evaluation, the computed tomography and magnetic resonance imaging findings described in this report suggest inclusion of this rare tumor in the differential diagnosis when such findings occur in the retroperitoneum.

## Introduction

Soft-tissue perineuriomas are rare benign peripheral nerve sheath tumors with perineurial cell differentiation [1-4]. They were first described by Lazarus and Trombetta in 1978 [1]. Although they occur most commonly in the subcutis of the extremities and the trunk of young to middle-aged adults, these lesions also infrequently occur in other locations. Four cases have been reported in the retroperitoneum with no computed tomography (CT) or magnetic resonance imaging (MRI) images [2,3].

To the best of our knowledge, this the first presentation of the CT and MRI images of a soft-tissue perineurioma in the retroperitoneum with pathologic correlation.

## Case presentation

**Figure 1:**
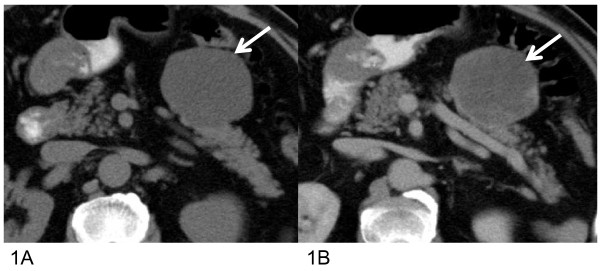
**Computed tomography results**. **(a) **Plain computed tomography (CT) shows a well-corcumscribed water-density (12HU) mass at the pancreatic body. **(b) **Contrast-enhanced CT shows the tumor with subtle enhancement (30HU).

**Figure 2:**
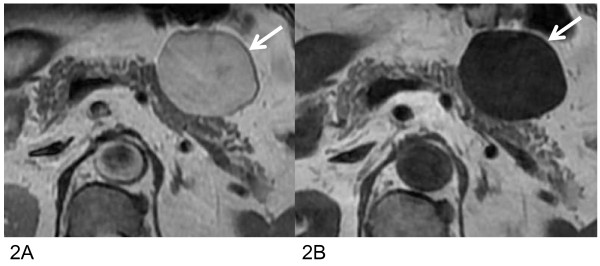
**Magnetic resonance imaging results**. **(a) **Axial T_2_-weighted MR image shows that the mass has almost homogeneous high signal intensity, **(b) **Axial T_1_-weighted MR images show that the mass has homogeneous low signal intensity.

**Figure 3:**
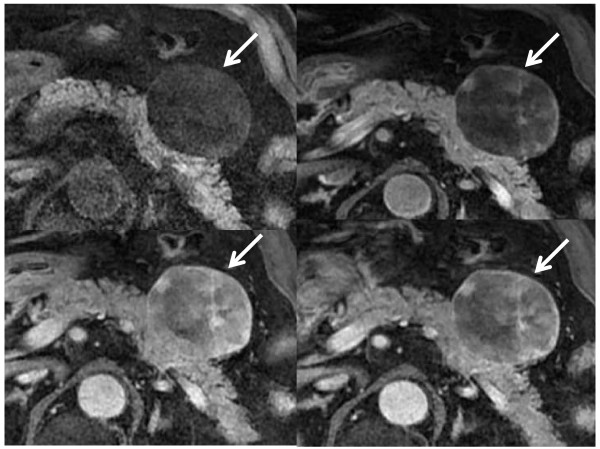
**Dynamic MR images show gradually increasing enhancement of the tumor after IV adminsitration of gadolinium-DTPA**. Pre-contrast (top left); post-contrast, 30 sec (top right); 60 sec (bottom left); and 90 sec (bottom right).

A 63-year-old Japanese man was admitted to our hospital for assessment of hypertension. Abdominal CT and MR imaging revealed a 4 cm well-defined mass at the anterior portion of the pancreatic body. The mass was hypodense with ca.12 HU on plain CT (Figure 1a), and only slightly enhanced on contrast-enhanced CT (Figure 1b). The mass was hyperintense on T_2_-weighted, and hypointense on T_1_-weighted MR images (Figure 2). Dynamic study using breath-hold gradient-echo with fat-suppression imaging after the administration of gadolinium-DTPA demonstrated gradually increasing signal enhancement of the entire mass (Figure 3). No central cystic degeneration, septum, or calcification was noted.

Tumor excision with distal pancretectomy and splenectomy was performed, as a malignant tumor of pancreatic origin could not be ruled out.

**Figure 4:**
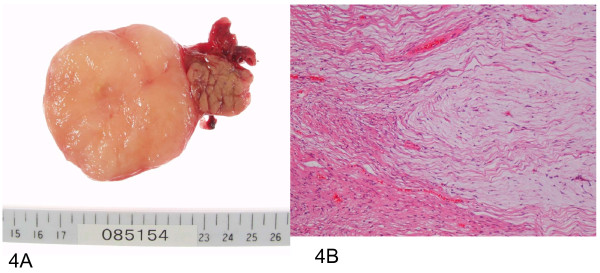
**Structure of the mass**. **(a) **Cut surface of a well-circumscribed, whitish, solid mass. **(b) **Hypocellular tumor composed of spindle cells with elongated nuclei and slender cytoplasmic processes in prominent myxoid stroma.

Macroscopically, the tumor was a well-circumscribed, whitish myxoid mass with no focal degenerative changes (Figure 4a). It was encapsulated with a fibrous capsule.

Microscopically, the tumor consisted of spindle cells with oval nuclei and thin bipolar elongated cytoplasmic processes loosely arranged in abundant myxoid stroma (Figure 4b).

The tumor was immunohistochemically characterized as a soft-tissue perineuroma, given its positive immunoactivity for CD 34, EMA, glut1, claudin-1, and negative immunoactivity for S-100. Ultrastructurally, the tumor cells possessed markedly attenuated bipolar cytoplasmic processes running in parallel, which are the characteristics of perineurial cells.

Although the tumor was firmly attached to the pancreas, no direct invasion was noted. Sixteen months after his operation, follow-up CT showed no evidence of a recurrent tumor.

## Discussion

Perineuriomas are uncommon benign peripheral nerve sheath tumors that include four subtypes: soft tissue, intramural, sclerosing, and reticular. Soft-tissue perineuriomas are the most common subtype and show distinctive morphologic, ultrastructual, and immunophenotypic features that distinguish them from the much more common nerve sheath tumors, schwannomas, and neurofibromas. The tumors show a morphologic spectrum ranging from hypercellular lesions with collagenous stroma to hypocellular tumors with myxoid stroma. The immunohistochemical studies are often necessary for the diagnosis of soft-tissue perineurioma [3,4].

To the best of our knowledge, MRI and CT images of soft-tissue perineurioma in the retroperitoneum have not been reported. The soft-tissue perineurioma in this report was similar to the one in the subcutis of the groin [5]: well-defined margins with fibrous capsule, water density on plain CT, subtle enhancement on contrast-enhanced CT, low signal on T_1_-weighted images, and hyperintensity with low-intensity capsule on T_2_-weighted images. Only contrast enhancement on fat-suppressed T_1_-weighted images was more prominent in our report. A dynamic MR study revealed the gradually enhancing nature of this myxoid tumor.

Nearly half of soft-tissue perineuriomas are hypocellular, and 20% are markedly hypercellular. The stroma can vary from a more collagenous to a myxoid appearance. Whereas the stroma in most tumors is collagenous, 40% show at least focally myxoid stroma, and 20% are nearly exclusively myxoid [2]. The soft-tissue perineurioma in our report was hypocellular with abundant myxoid stroma.

Radiologic differential diagnosis of retroperitoneal soft-tissue perineurioma includes neurilemmomas, neurofibromas [6], ganglioneuromas [7], and cellular myxomas [8]. The former two tumors are most common nerve sheath tumors, and a target-like enhancement pattern is the MR imaging characteristic. Moreover, they tend to demonstrate multiple cystic spaces of varying size that are caused by either cystic or myxoid degeneration within the tumors. Hemorrhage and calcification may be also seen [6].

In contrast, the current case of soft-tissue perineurioma showed no definite focal degeneration or hemorrhagic areas. Cellular/intramuscular myxomas arise in the muscle, and are rarely found in the retroperitoneum [8]. The most problematic differentiation may be between soft-tissue perineurioma and ganglioneuroma with abundant myxoid stroma. They both show rather homogeneous marked hyperintensity on T_2_-weighted images. The tumor signal intensity on T_2_-weighted images depends on the proportion of myxoid stroma to cellular components and collagen fibers. MR dynamic enhancement patterns of the two entities are similar: they lack early enhancement, but delayed enhancement increased gradually. However, one of the MR imaging characteristics of ganglioneuroma is curvilinear bands of low signal intensity on T_2_-weighted images [7], which were absent in the current soft-tissue perineurioma. The soft-tissue perineuromas can be either encapsulated or unencapsulated [2]. Neurilemmomas are encapsulated, and neurofibromas are unencapsulated tumors [6], and the possibility of neurofibroma can be excluded if the findings suggest the presence of a capsule [7].

Other differential diagnoses include malignant tumors such as perineurial malignant nerve sheath tumor [9], malignant fibrous histiocytoma with myxoid change, and low-grade fibromyxoid sarcoma.

Dynamic MR imaging better provides the gradually enhancing nature of a myxoid tumor. Soft-tissue perineuriomas with marked myxoid stroma may be mistaken for cysts on plain CT.

## Conclusion

Although the definitive diagnosis of soft-tissue perineurioma requires biopsy and immunohistochemical reactivity evaluation, the CT and MRI findings described in this report suggest inclusion of this rare tumor in the differential diagnosis when such findings occur in the retroperitoneum.

## Competing interests

The authors declare that they have no competing interests. Funding was neither sought nor obtained.

## Authors' contributions

MY conceived the study. YK and RM performed the literature review. AA and KK performed histopathologic and immunohistochemical analyses. All authors read and approved the final version of the manuscript.

## Consent

Written informed consent was obtained from the patient for publication of this case report and accompanying images. A copy of the written consent is available for review by the Editor-in-Chief of this journal.
